# Drop jump performance differences between ACL-injured and healthy semi-professional male soccer players

**DOI:** 10.3389/fspor.2025.1618284

**Published:** 2025-07-16

**Authors:** Dimitrije Cabarkapa, Damjana V. Cabarkapa, Yu Song, Andrew C. Fry, Thordis Gisladottir, Milos Petrovic

**Affiliations:** ^1^ Jayhawk Athletic Performance Laboratory, Wu Tsai Human Performance Alliance, Department of Health, Sport and Exercise Sciences, University of Kansas, Lawrence, KS, United States; ^2^ D2 Lab, Novi Sad, Serbia; ^3^ Faculty of Physical Culture and Sport Management, Singidunum University, Belgrade, Serbia; ^4^ Biomechanics Laboratory, Department of Health, Sport and Exercise Sciences, University of Kansas, Lawrence, KS, United States; ^5^ Research Center for Sport and Health Sciences, University of Iceland, Reykjavik, Iceland

**Keywords:** force, asymmetry, concentric, eccentric, recovery, rehabilitation, return-to-play

## Abstract

The purpose of this study was to examine differences in lower-body neuromuscular performance characteristics between ACL-injured athletes and their healthy counterparts, including peak take-off and landing force asymmetries. Forty-four semi-professional male soccer players volunteered to participate in the present investigation, from which 16 had previously undergone ACL reconstruction procedures and 28 were healthy controls. Following the warm-up completion, athletes performed three non-consecutive drop jumps (30 cm) with no arm swing while landing on a uni-axial force plate system sampling at 1,000 Hz. The injured athletes were screened nine months post-operative procedures and all athletes were active members of their respective soccer teams. The dependent variables included the force-time metrics within both the eccentric and concentric phases of the drop jump. Independent t-tests or Mann–Whitney U-tests were used to examine statistically significant (*p* < 0.05) differences in each variable (ACL-injured vs. healthy controls). The results revealed that ACL-injured athletes tend to display significantly lower jump heights (39.4%), shorter eccentric duration (21.1%), and greater peak drive-off force asymmetries (50.6%) when compared to their non-injured counterparts. Also, despite not reaching the level of statistical significance and being small-to-moderate in magnitude, ACL-injured participants attained shorter contact times (8.8%) and greater peak impact force asymmetry (16.1%).

## 1 Introduction

Soccer is one of the most popular international sports that entails high physical and physiological demands (1), with players covering approximately 10–12.5 km during a single match (2). Besides the total distance covered at various intensities, accelerations/decelerations, change-of-direction, and landing maneuvers are commonly embedded in the game of soccer (3, 4). For example, the most commonly performed movement preceding a goal is a forward sprinting motion followed by rapid deceleration and change-of-direction (4). However, these movement patterns have been positively associated with an increased incidence of anterior cruciate ligament (ACL) injury events in male soccer players (5). While the ACL injury mechanism has been well-documented over the last two decades (6), the overall injury risk in soccer has remained unchanged (7). So, when taking into account the long-term negative consequences of ACL injuries (1, 8), including the reinjury risk (9), a deeper understanding of physiological performance characteristics (e.g., neuromuscular performance) following ACL injury in soccer players is of critical importance.

Bilateral compensatory strategies are commonly observed in patients after ACL injuries, particularly during jump-landing maneuvers (10–13). For instance, more than 10% of bilateral asymmetries in vertical ground reaction forces were found within both concentric and eccentric phases of countermovement vertical jump (CMJ) (12, 13). Precisely, considerably lower vertical ground reaction forces were observed in the ACL-injured vs. non-injured limb within a cohort of collegiate athletes (12, 13). Similarly, in male soccer players, a significant reduction in vertical ground reaction force and impulse has been reported in the ACL-injured limb within both eccentric and concentric phases of the CMJ when compared to the non-injured limb, as well as when compared to a group of healthy non-injured counterparts (11). However, despite CMJ being a simple and widely utilized functional weight-bearing task to assess movement patterns and lower-body force and power-producing capabilities (14–16), recent findings suggest that the CMJ may not be sufficiently demanding to detect persistent lower-limb movement deficits in soccer players even more than 11 months post-operative procedures (17). In addition, ACL injuries frequently occur during sudden deceleration movements, which further emphasizes the importance of eccentric force-producing capabilities (5, 18). Thus, in an applied sport-specific setting, the drop jump has been proposed as a more appropriate task for assessing bilateral asymmetries in ACL patients, as it more closely replicates the rapid deceleration and landing maneuvers (e.g., eccentric muscle contraction) associated with ACL injury risk patterns observed in the game of soccer (10).

To date, several studies have investigated bilateral asymmetries during drop jumps following ACL injuries (10, 19, 20). For instance, more asymmetrical peak impact forces, smaller in magnitude, were found during the drop jump test in ACL-injured individuals compared to healthy controls (20). Also, ACL-injured adolescents demonstrated lower jump height, greater inter-limb force production asymmetry, and peak impact force in comparison to their healthy counterparts (10). However, no significant group differences have been observed in contact time or within the eccentric and concentric phases of CMJ (10). Moreover, previous research found no differences in peak impact force between ACL-injured and healthy individuals within a group of collegiate athletes (19). Therefore, the current inconsistencies among various populations highlight the need for a better understanding of drop jump performance in ACL-injured individuals, particularly in male soccer players who are predisposed to an increased incidence of ACL injury through their competitive career (21), to improve rehabilitation strategies and return-to-play assessments.

Based on the currently available scientific literature and the knowledge gap, the purpose of the present study was to examine differences in lower-body force-time metrics between ACL-injured semi-professional male soccer players and their healthy counterparts, including take-off (i.e., concentric) and landing (i.e., eccentric) asymmetries in force production during a drop jump activity. Based on the previously published literature, it is hypothesized that the drop jump test would provide a sufficient stimulus for detecting between-group performance differences.

## 2 Materials and methods

### 2.1 Participants

Forty-four semi-professional male soccer players volunteered to participate in the present investigation, from which 16 were ACL-injured (age = 24.1 ± 4.7 years; body mass = 81.6 ± 9.8 kg; height = 182.7 ± 5.7 cm) and 28 non-injured (age = 21.7 ± 3.5 years; body mass = 77.4 ± 10.7 kg; height = 179.4 ± 8.3 cm) healthy athletes (i.e., control group). Participants were included in this study if they met the following criteria: (i) aged 18–30 years, (ii) sustained a complete ACL tear, (iii) underwent ACL reconstruction, (iv) completed a standardized nine-month rehabilitation protocol, (v) cleared by medical staff to participate in team training activities. Exclusion criteria were as follows: (i) history of additional knee injuries beyond an ACL tear and (ii) prior knee surgery (e.g., meniscus or medial collateral ligament repair). The control group encompassed athletes competing at the same level of play with no history of knee injury (e.g., ACL tear) and were active members of the semi-professional soccer team. To ensure consistency in surgical procedures and rehabilitation protocols, all the data was obtained from the same clinic. The study was approved by the University's Institutional Review Board, and all participants provided written informed consent before participation.

### 2.2 Procedures

Prior to the start of the testing procedures, all athletes completed a standardized warm-up consisting of a low-intensity run on a treadmill (i.e., 3–5 min) and a set of dynamic stretching exercises (e.g., high-knees, butt-kicks, A-skip). Following completion of the warm-up protocol, the athlete stepped on a wooden box (30 cm) positioned directly above the uni-axial force plate (ForceDecks Max, VALD Performance, Brisbane, Australia), sampling at 1,000 Hz. On the “go” command (i.e., 3-2-1-go), the athlete stepped off the box onto the force plates, quickly absorbing the landing impact (i.e., eccentric action), and immediately transitioning into a maximal-effort vertical jump (i.e., concentric action). Also, the athletes were instructed to focus on attaining minimal ground contact time (e.g., jump off the ground as quickly as possible, like landing on a hot surface) (22). The athletes were instructed to jump and land at approximately the same spot on the force plates and keep their hands on their hips during the entire movement (i.e., no arm swing). Each athlete completed a total of three jump trials, with the average value being used for performance analysis purposes. To minimize the possible influence of fatigue, each jump was separated by a 30–45 s rest interval.

The force-time metrics of interest examined in the present study were the following: jump height (i.e., maximum vertical displacement of the center of mass between takeoff and landing), contact time, reactive strength index (i.e., jump height measured via flight time calculation divided by the contact time; RSI), eccentric and concentric duration, and peak impact and drive-off and landing force (i.e., relative to athlete's body mass). Based on previously published research reports, these variables have demonstrated strong levels of validity and reliability for assessing lower-body neuromuscular performance characteristics (15, 23–25). In addition, the inter-limb peak impact and drive-off force asymmetries were calculated (i.e., [(right limb-left limb)/ ½ (right limb + left limb)] × 100%) (26). The drop landing was determined when the system mass exceeded the 20N threshold. The peak impact force was represented as the greatest passive force on the impact from the box drop (30 cm). The start of the concentric phase was defined as the upward movement where the athlete quickly accelerated after a brief landing on the force plates by extending their legs and rapidly pushing off the ground. Lastly, the peak drive-off force was characterized as the maximal active force (contraction-based force) observed during the concentric phase of the jumping movement. If needed, the detailed explanation can be found at the following website: https://valdhealth.com/news/understanding-the-drop-jump.

### 2.3 Statistical analysis

Shapiro–Wilk tests and Q-Q plots were used to examine the assumption of normality. Independent t-tests (mean and standard deviation) or Mann–Whitney U-tests (mean and interquartile range) were used to examine statistically significant between-group differences (i.e., ACL-injured vs. healthy), depending on whether the variable met or violated the assumption of normality, respectively. For normally distributed variables the effect sizes (Hedges' g) were calculated by dividing the mean difference between the two groups with the pooled standard deviation (i.e., *g* < 0.2—small; *g* = 0.2–0.5—medium; *g* > 0.8 large) and for non-normally distributed variables the effect sizes were calculated by dividing the Z-statistic with the square root of the sample size (i.e., *r* = Z/√N; *r* < 0.3 small; *r* = 0.3–0.5—medium; *r* > 0.5—large) (27, 28). The percent difference between two values was calculated by determining the absolute difference, dividing it by the mean of the two values, and multiplying the result by 100. The *α* level of *p* < 0.05 was used as a criterion for statistical significance. All statistical analysis procedures were completed in SPSS (Version 28.0; Chicago, IL, USA).

## 3 Results

Descriptive data and statistical comparisons for each dependent variable, including the effect sizes, can be found in Table 1. Between-group statistically significant differences were found in jump height, eccentric duration, and peak drive-off force asymmetry. Healthy non-injured athletes attained considerably greater jump heights (39.4%) when compared to their ACL-injured counterparts, while displaying longer times (21.1%) spent within the eccentric phase of the drop jump movement. Also, peak drive-off asymmetry was pronounced to a considerably greater extent (50.6%) in ACL-injured athletes than within healthy athletes (control group). Additionally, no statistically significant difference between the groups was found in body mass (*p* = 0.294), stature (*p* = 0.303), and age (*p* = 0.143).

**Table 1:** Descriptive statistics, mean and standard deviation ($\bar{x}$ ± SD) or median and interquartile range (M[IQR]), for drop jump performance variables.

Variable [unit]	Healthy	ACL-injured	*p*-value [ES]
Jump height [cm]	38.0 ± 5.9	25.5 ± 8.7*	<0.001 [1.778]
Contact time [sec]	0.379 ± 0.084	0.347 ± 0.098	0.282 [0.359]
RSI [ratio]	1.54 [0.50]	1.55 [0.96]	0.798 [0.039]
Eccentric duration [sec]	0.210 ± 0.045	0.170 ± 0.040*	0.004 [0.924]
Concentric duration [sec]	0.169 ± 0.041	0.177 ± 0.065	0.669 [0.157]
Peak impact force [N/kg]	46.8 [25.8]	40.6 [20.0]	0.558 [0.088]
Peak drive-off force [N/kg]	29.7 [9.8]	34.2 [13.7]	0.227 [0.182]
Peak impact force ASY [%]	17.7 ± 10.8	20.8 ± 14.1	0.378 [0.257]
Peak drive-off force ASY [%]	9.0 ± 6.3	15.1 ± 10.4*	0.048 [0.762]

* Significantly different when compared to healthy group of athletes (*p* < 0.05).

ES, effect size (Hedge's g); ACL, anterior cruciate ligament; RSI, reactive strength index.

## 4 Discussion

The purpose of the present study was to examine differences in lower-body neuromuscular performance characteristics between ACL-injured semi-professional male soccer players and their healthy counterparts (i.e., control group). The findings generally supported the aforementioned hypothesis as multiple between-group differences in force-time metrics were observed. Specifically, significantly lower jump height (39.4%), shorter eccentric duration (21.1%), and greater peak drive-off force asymmetry (50.6%) were found in ACL-injured than healthy athletes. Also, while not reaching the level of statistical significance, it is worth noting that small-to-moderate effect size differences (*g* = 0.257–0.325) were observed in contact time and peak impact force asymmetry. The ACL-injured participants attained shorter contact times (8.8%) and greater peak impact force asymmetry (16.1%).

In an applied sport setting, drop jump and its variations (e.g., bilateral or unilateral) have been widely used as a valid and reliable field-based test for the assessment of ACL-injury risk and athletes' return-to-play readiness status (29–35). For example, Kostifaki et al. (31) found that jump height was significantly lower in injured than non-injured athletes, which aligns with the results obtained in the present investigation. Similarly, when studying a cohort of collegiate athletes, Lem et al. (19) found that participants who underwent ACL reconstruction attained 21.6% lower jump heights than their non-injured teammates matched by activity level, age, sex, and anthropometric characteristics. Also, the presence of this post-operative performance decrement in drop jump height was noted in multiple research reports, especially within the 7–10-month period (19, 30, 32), which matches with the testing timeline implemented in this study (i.e., nine months post-ACL reconstruction). However, previous research has also found that the decrease in jump height was commonly accompanied by a reduction in RSI (30–32). These findings are contradictory to the results obtained in the present investigation, as no difference in RSI between injured and non-injured semi-professional soccer players was noted (<1%). While further research is warranted on this topic, this discrepancy could be attributed to the complexity of the drop jump testing procedures (e.g., unilateral vs. bilateral jumps and box height), training status (e.g., recreational active individuals vs. athletes), as well as the sex-specific biomechanical differences (e.g., increased knee valgus and higher ground reaction forces) (36–38).

The aforementioned biomechanical differences could also contribute to shorter eccentric durations (21.1%) observed within ACL-injured athletes compared to their healthy counterparts (33, 39, 40). For instance, during the eccentric phase of the drop jump movement, the muscles are actively lengthening to absorb the impact, which lasts from the initial contact with the ground until the body reaches zero velocity (40, 41). Previous research has found that female athletes who underwent ACL reconstruction tend to display altered landing kinematics than their non-injured teammates (35). Specifically, ACL-injured athletes had a significant reduction in peak knee flexion (62.0 vs. 69.5 deg) and adduction (3.0 vs. 8.9 deg) compared to non-injured athletes (35). Thus, we can assume that these biomechanical alterations did not allow athletes to adequately absorb the landing impact (e.g., stiffer landing), which ultimately resulted in shorter time spent within the eccentric phase of the jumping movement (11). Also, similar changes in jump kinematics were observed in soccer players who did not even sustain an ACL tear but had a history of lower-body injury, resulting in increased knee valgus angle and decreased knee flexion (33). In addition, despite not reaching the level of statistical significance, it should be noted that overall contact times observed in the present study were slightly longer (8.8%) in healthy than in ACL-injured athletes. When considering that trivial between-group differences were observed in concentric duration, we can assume that the decrease in eccentric duration caused by alterations in landing biomechanics could be one of the key factors that caused this slight decrement in the overall contact time.

Besides being focused on analyzing the jump height, RSI, and contraction times, asymmetries in force production need to be incorporated into the analysis procedures to obtain a comprehensive insight into athletes' readiness or return-to-play status. For example, a recently published study indicated that individuals after ACL reconstruction displayed asymmetries in relative muscle contribution 10 months post-operative procedures, implying altered muscle coordination strategies (31). While asymmetries are prevalent in team sports such as basketball, volleyball, and handball, they do not seem to impair an athlete's performance (42). Still, certain asymmetries and alterations in movement patterns that seem to be absent before injury and tend to appear in response to the injury (e.g., knee-extension moment) could be an issue if they remain unresolved (39, 42). These research reports align with the findings obtained in the present investigation. For example, the asymmetries in peak impact force were present in both ACL-injured and healthy athletes and were not significantly different between the groups (16.1%). However, when examining the asymmetries in peak drive-off force during the concentric phase of the drop jump, notable differences were observed, with ACL-injured athletes demonstrating greater asymmetry magnitudes (50.6%). While further research is warranted on this topic, a possible explanation can be the implementation of compensatory offloading strategies used to protect the injured limb during various athletic performance tasks such as drop jump (11). Also, it has been found that these asymmetries in force-producing capabilities may persist even after nine-month-long rehabilitation programs, which further solidifies the importance of longitudinal athlete tracking and obtaining baseline data (i.e., pre-injury data) to objectively quantify the athletes' progress over time (11, 43).

While providing a deeper insight into the difference in lower-body neuromuscular performance characteristics between ACL-injured and healthy semi-professional soccer players, this study is not without limitations. The lack of pre-injury data for each athlete (e.g., baseline) is one of the main limitations of this investigation, as this type of data could provide detailed pre-post-performance assessment on a within-subject basis. Also, the cohort of participants was relatively uniform, comprising only semi-professional soccer players competing at a similar level of play. So, further research is warranted to explore whether these findings are consistent across different competitive levels (e.g., collegiate, professional) and various team and individual sports (e.g., basketball, tennis), as well as if they are sex-specific.

In conclusion, the results of the present study revealed that ACL-injured athletes tend to display significantly lower jump heights (39.4%), shorter eccentric duration (21.1%), and greater peak drive-off force asymmetry (50.6%) when compared to their healthy non-injured counterparts. Overall, these findings highlight the effectiveness of the drop jump test performed on a dual uni-axial force plate system as a simple and non-invasive testing method that can provide a deeper insight into athletes' lower-body neuromuscular performance characteristics, including force-producing asymmetry measures. This can help coaches, sports scientists, and strength and conditioning practitioners identify performance deficiencies that may predispose athletes to greater injury risk as well as help optimize post-injury recovery progress and return-to-play readiness.

## Data Availability

The raw data supporting the conclusions of this article will be made available by the authors, without undue reservation.
